# G1T48, an oral selective estrogen receptor degrader, and the CDK4/6 inhibitor lerociclib inhibit tumor growth in animal models of endocrine-resistant breast cancer

**DOI:** 10.1007/s10549-020-05575-9

**Published:** 2020-03-04

**Authors:** Kaitlyn J. Andreano, Suzanne E. Wardell, Jennifer G. Baker, Taylor K. Desautels, Robert Baldi, Christina A. Chao, Kendall A. Heetderks, Yeeun Bae, Rui Xiong, Debra A. Tonetti, Lauren M. Gutgesell, Jiong Zhao, Jessica A. Sorrentino, Delita A. Thompson, John E. Bisi, Jay C. Strum, Gregory R. J. Thatcher, John D. Norris

**Affiliations:** 1 Department of Pharmacology and Cancer Biology, Duke University School of Medicine, 308 Research Drive, Durham, NC 27710 USA; 2 G1 Therapeutics, Inc, 700 Park Offices Drive, Suite 200, Research Triangle Park, NC 27709 USA; 3 Department of Pharmaceutical Sciences, College of Pharmacy, University of Illinois at Chicago, 833 South Wood Street (M/C 781), Chicago, IL 60612 USA

**Keywords:** G1T48, Selective estrogen receptor degrader, Endocrine-resistant breast cancer, CDK4/6 inhibitor, Combination therapies

## Abstract

**Purpose:**

The combination of targeting the CDK4/6 and estrogen receptor (ER) signaling pathways with palbociclib and fulvestrant is a proven therapeutic strategy for the treatment of ER-positive breast cancer. However, the poor physicochemical properties of fulvestrant require monthly intramuscular injections to patients, which limit the pharmacokinetic and pharmacodynamic activity of the compound. Therefore, an orally available compound that more rapidly reaches steady state may lead to a better clinical response in patients. Here, we report the identification of G1T48, a novel orally bioavailable, non-steroidal small molecule antagonist of ER.

**Methods:**

The pharmacological effects and the antineoplastic mechanism of action of G1T48 on tumors was evaluated using human breast cancer cells (in vitro) and xenograft efficacy models (in vivo).

**Results:**

G1T48 is a potent and efficacious inhibitor of estrogen-mediated transcription and proliferation in ER-positive breast cancer cells, similar to the pure antiestrogen fulvestrant. In addition, G1T48 can effectively suppress ER activity in multiple models of endocrine therapy resistance including those harboring ER mutations and growth factor activation. In vivo, G1T48 has robust antitumor activity in a model of estrogen-dependent breast cancer (MCF7) and significantly inhibited the growth of tamoxifen-resistant (TamR), long-term estrogen-deprived (LTED) and patient-derived xenograft tumors with an increased response being observed with the combination of G1T48 and the CDK4/6 inhibitor lerociclib.

**Conclusions:**

These data show that G1T48 has the potential to be an efficacious oral antineoplastic agent in ER-positive breast cancer.

**Electronic supplementary material:**

The online version of this article (10.1007/s10549-020-05575-9) contains supplementary material, which is available to authorized users.

## Introduction

The estrogen receptor (ER/*ESR1*) is expressed in a majority of breast cancers, and drugs that inhibit ER signaling are the cornerstone of breast cancer pharmacotherapy for ER-positive/HER2-negative disease [1]. These targeted approaches include the Selective Estrogen Receptor Modulator (SERM) tamoxifen that acts as a competitive ER antagonist in the breast, and aromatase inhibitors (AIs) that inhibit aromatase, the enzyme responsible for estrogen production [2, 3]. However, the development of resistance limits the duration of meaningful therapeutic responses. Mechanisms of resistance to these endocrine therapies include cell cycle dysregulation and activation of alternative growth factor signaling pathways [1]. For example, activation of MAPK, PI3K, and GSK-3 can result in increased phosphorylation of ER or its attendant coregulatory proteins leading to ligand-independent ER activity and resistance [4–7]. Recently, genomic alterations in the ER gene itself, including amplification, translocation, and ligand binding domain mutations (most frequently ER-D538G and ER-Y537S) have emerged with AI therapy [1, 8–10].

After progression during tamoxifen and AI therapy, other endocrine treatments including the steroidal selective estrogen receptor downregulator (SERD) fulvestrant (Faslodex^®^) are generally used [11]. SERDs are a class of ER antagonists that in addition to competitively displacing estrogens, also trigger ER downregulation [12]. Although initially successful, the onset of resistance limits durable responses when used as a monotherapy. Therefore, in an effort to improve the therapeutic lifespan of endocrine treatments for metastatic breast cancer, combination regimens have been extensively studied. Clinical trials using a combination of AI or fulvestrant with pan-PI3K or mTOR inhibitors have been promising but inconclusive, and toxicity often remains an impediment to dose escalation [13–16]. Therefore, CDK4/6 inhibitors have emerged as a favored option when considering combination endocrine therapies [17–20]. However, the poor bioavailability of fulvestrant, coupled with its intramuscular route of administration and the long time to steady state blood levels, compromises its clinical use [21, 22]. Indeed, even at the higher clinical dose (500 mg) of fulvestrant, pharmacodynamic imaging suggests incomplete receptor saturation [23].

Collectively, these data highlight an unmet need for a safe, orally bioavailable SERD with appropriate pharmaceutical properties. Herein we describe the preclinical development of G1T48 (rintodestrant), an orally bioavailable, potent, and selective non-steroidal ER antagonist and downregulator [24]. G1T48 was found to robustly inhibit ER activity in multiple in vitro models of endocrine therapy resistance, including those harboring ER mutations or growth factor activation. Importantly, G1T48 demonstrated robust antitumor activity in an animal model of early stage estrogen-dependent breast cancer and suppressed the growth of tamoxifen- and estrogen deprivation-resistant xenograft tumors with increased efficacy observed for the combination of G1T48 and lerociclib, a newly developed CDK4/6 inhibitor [25, 26].

## Methods

### Reagents

Fulvestrant (CAS No: 129453–61-8, > 99% purity) was purchased from MedChem Express. Estradiol (E2) (E8875), lasofoxifene (SML1026), 4-hydroxytamoxifen (H7904), and tamoxifen (T5648) were purchased from Sigma. Raloxifene (2280) was purchased from Tocris. GDC-0810 (S7855), bazedoxifene (S2128), and AZD9496 (S8372) were purchased from Selleckchem. GW5638 (5638), GW7604 (7604), and RU 58,668 (RU) were provided by Donald McDonnell (Duke University). G1T48 was provided by G1 Therapeutics, Inc., as analytical grade compound.

### RNA analysis

MCF7 cells were authenticated by short tandem repeat profiling, were tested for *Mycoplasma* and were not cultured for more than three months at a time [27]. MCF7 cells were plated in DMEM/F12 supplemented with 8% charcoal dextran treated FBS for 48 h. Cells were then treated for 24 h with ligand and RNA was isolated using the Aurum™ total RNA isolation kit (Bio-Rad, Hercules, CA). After cDNA synthesis (iScript kit, Bio-Rad) real-time PCR was performed using the Bio-Rad CFX384 real-time system. GAPDH mRNA expression was used to normalize all real-time data using the 2-ΔΔC_T_ method [28]. For more detailed description of this method, please see Online Resource 1.

### Proliferation

MCF7 cells were plated in DMEM/F12 supplemented with 8% charcoal dextran treated FBS in 96-well plates (5 K cells/well) for 48 h. Cells were treated with estradiol (0.1 nM) or insulin (20 μM) with or without test compound (dose response; 1.0^–11^ to 1.0^–05 ^M) for 6 days. Plates were decanted and frozen at – 80°°C overnight prior to quantitation of DNA by fluorescence using Hoechst 33258.

### Supplementary material

Detailed methods are available in Online Resource 1 for the following protocols: In-Cell Western, Radioactive Binding Assay, Chromatin Immunoprecipitation, Transcriptional Reporter Assays.

### Murine studies

All procedures were approved by the Institutional Animal Care and Use Committee (IACUC) of Duke University or South Texas Accelerated Research Therapeutics (START, San Antonio, Texas) prior to initiating the experiment. For complete details, see Online Resource 1.

## Results

### G1T48 is similar to fulvestrant in its ability to downregulate the estrogen receptor and inhibit estrogen signaling in breast cancer cells

**Fig. 1:**
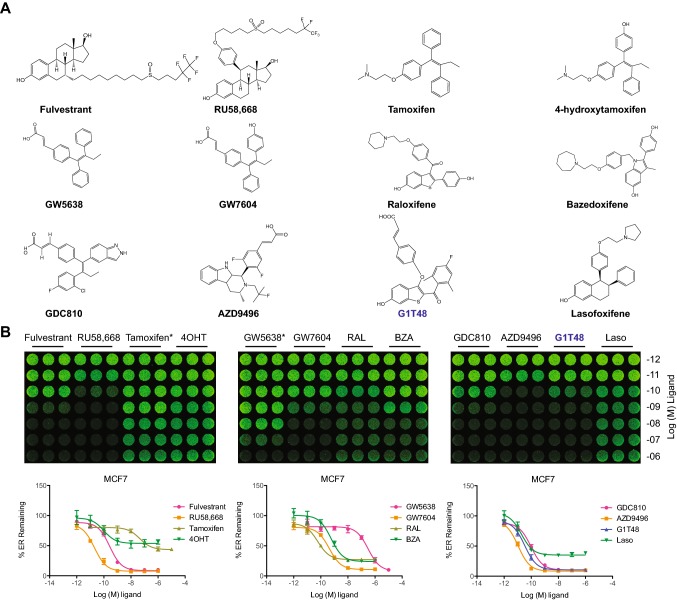
G1T48 is a potent selective estrogen receptor downregulator (SERD). **a** Chemical structures of G1T48 and benchmark SERMs and SERDs. **b** G1T48 downregulates the estrogen receptor in breast cancer cells. MCF7 cells were treated with ER ligands (10^–12^–10^–6^ M) for 18 h prior to fixation and detection of ER levels by In-Cell Western. *For tamoxifen and GW5638, dose response was 10^–11^–10^–5^ M. Error bars indicate the SD of triplicate samples

Novel ER antagonists with SERD activity have recently been described, but clinical development of these compounds has thus far been limited due to unanticipated side effects or for undisclosed reasons [29–36]. We sought to identify an orally bioavailable SERD using the chemical backbone of raloxifene as a starting point, since this SERM has demonstrated a favorable safety profile in the clinical setting of breast cancer prevention and osteoporosis treatment [37, 38]. G1T48 incorporates an acrylic acid side chain (Fig. 1a) [29, 31, 32, 34, 39, 40], and was the product of structure-guided investigations, driven by activity in breast cancer cell lines [24]. G1T48 was first assessed for its ability to downregulate ER when compared to several benchmark SERMs and SERDs including fulvestrant [12, 41]. Using In-Cell Western assays, G1T48 was found to downregulate ER with an efficacy modestly more potent than steroidal and other SERDs (e.g., fulvestrant, AZD9496; approximately 10% ER remaining after treatment) (Fig. 1b, online resource 2). Bazedoxifene (BZA), raloxifene (RAL), tamoxifen, 4-hydroxytamoxifen (4OHT), and lasofoxifene (laso) were also found to partially downregulate ER. These data demonstrate that in vitro G1T48 is a pure antiestrogen and selective estrogen receptor degrader (PA-SERD).

**Fig. 2:**
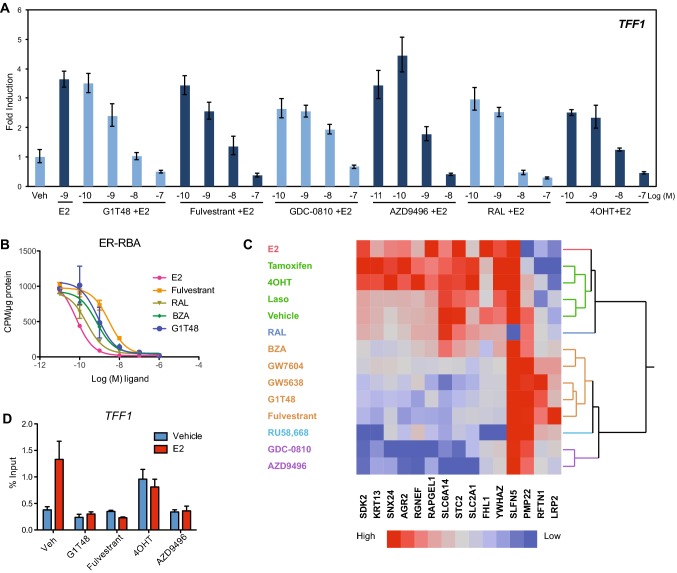
G1T48 is a complete estrogen receptor antagonist. **a** G1T48 inhibits ER target gene expression in breast cancer cells. MCF7 cells were treated with ER antagonists (10^–10^–10^–7^ M) plus estradiol (E2; 10^–9^ M) for 18 h. *TFF1* mRNA expression was analyzed by real-time PCR. GAPDH was used to normalize real-time PCR data. **b** G1T48 competes for estrogen binding to ER. MCF7 cells were treated with 10^–10^ M ^3^H-17β-E2 and competitor ligand (10^–12^–10^–6^ M) for 2 h. Cells were collected and radioactive counts were monitored on a Beckman LS 6000SC Scintillation counter. Error bars indicate the SD of duplicate samples. **c** G1T48 regulates ER target gene pharmacology similar to other SERDs. MCF7 breast cancer cells were treated with ER ligands (E2, fulvestrant, RU, RAL @ 100 nM; G1T48, 810, 9496, Laso, 4OHT, 7604, BZA @ 1.0 μM; 5638, Tam @ 10 μM) for 24 h. mRNA expression was analyzed by real-time PCR. GAPDH was used to normalize real-time PCR data. Heatmaps were generated from real-time PCR data after analysis with JMP pro software (SAS) using the Ward hierarchical clustering algorithm. **d** G1T48 blocks estrogen-dependent recruitment of ER to the *TFF1* promoter**.** MCF7 cells were treated with ligand (E2: 5 × 10^−10^ M; ER antagonists: 10^–6^ M) as indicated for 45 min. Cells were fixed with formaldehyde and chromatin was immunoprecipitated with anti-ER antibody. Real-time PCR was used to assess the relative amount of ER bound to the *TFF1* gene promoter. Error bars indicate the SD of triplicate samples

We next evaluated the ability of G1T48 to inhibit endogenous ER target gene transcription in MCF7 cells. As shown in Fig. 2a, G1T48 suppressed estrogen-mediated activation of the Trefoil Factor-1 (*TFF1*) mRNA similarly to fulvestrant and additional antiestrogens (4OHT, GDC-0810, AZD9496, RAL). The biochemical basis of G1T48-mediated ER antagonism was further evaluated using ^3^H-estradiol whole-cell competition assay. Results showed that G1T48 displaced radiolabeled agonist binding with potency greater than fulvestrant and similar to BZA (Fig. 2b). Radioligand binding assay (RBA) IC_50_ shows that G1T48 is a competitive ER antagonist (Online Resource 3).

The inability of some ER antagonists, notably SERMs, to completely oppose the actions of estradiol is seen as a liability when being considered for the treatment of advanced therapy-resistant breast cancer. While data from Fig. 1b confirm G1T48 is a SERD, the potential remains for SERM activity, as G1T48 was developed based on compounds that exhibit both SERM and SERD activity. Therefore, we next considered the impact of G1T48 treatment on ER target genes that are differentially regulated by SERMs and SERDs [42]. As shown in Fig. 2c, compounds with SERM activity regulate these genes in a manner similar to the agonist estradiol, a reflection of their intrinsic agonist potential (red, green, and blue clusters). In contrast, G1T48 regulates these genes in a pattern that is consistent with compounds previously shown to downregulate ER (e.g., GW7604, fulvestrant, GW5638, RU 565899, GDC-0810, and AZD9496; orange, teal and purple clusters).

When bound by estrogen, ER is recruited to target gene promoters to activate or repress target gene transcription through recruitment of coregulator (coactivator or corepressor) proteins that modify chromatin structure [43]. We assessed the ability of G1T48 and benchmark SERMs or SERDs to inhibit estrogen-mediated recruitment of ER to the *TFF1* promoter using chromatin immunoprecipitation (ChIP) assays. While estrogen and 4OHT treatment significantly increased ER binding to the *TFF1* promoter (Fig. 2d), G1T48 inhibited the binding of ER to this promoter, with or without estrogen, with efficacy similar to fulvestrant, supporting the idea that G1T48 is an efficient ER antagonist in vitro.

We next evaluated the ER selectivity of G1T48 by assessing its ability to inhibit the transcriptional activity of related steroid hormone receptors androgen receptor (AR), progesterone receptor (PR), glucocorticoid receptor (GR), and mineralocorticoid receptor (MR) using a cell-based reporter assay. When administered at doses up to 10 µM, G1T48 did not affect the transcriptional activities of AR, GR, MR, or PR (Online Resource 4), indicating that G1T48 is a highly selective antagonist of ER.

### G1T48 inhibits the growth of ER-positive breast cancer cells

**Fig. 3:**
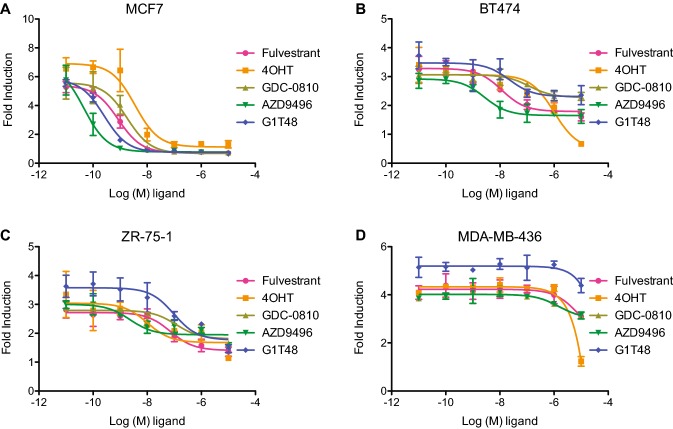
G1T48 inhibits ER-positive breast cancer cell growth. **a** ER-positive MCF7, **b** ER-positive BT474 **, c** ER-positive ZR-75–1 were treated for 7 days with 10^–10^ M E2 in addition to ER antagonists (10^–11^–10^–5^ M). **d** ER-negative MDA-MB-231 cells were treated for 7 days with ER antagonists (10^–11^–10^–5^ M). Cellular proliferation was assessed by measuring DNA content (Hoechst stain) and is presented as fold induction over vehicle control. Error bars indicate the SD of triplicate samples

To examine the therapeutic potential of G1T48, we performed cell proliferation assays using multiple ER-positive breast cancer cell lines (Fig. 3). G1T48 significantly inhibited estrogen-mediated growth of MCF7 cells demonstrating approximately threefold higher potency when compared to fulvestrant (Fig. 3a, Online Resource 5). Additionally, G1T48 and benchmark antiestrogens also inhibited the estrogen-mediated growth of ER-positive BT474 and ZR-75-1 breast cancer cells, while no growth inhibition was observed in ER-negative MDA-MB-436 breast cancer cells (Fig. 3, Online Resource 5). Furthermore, G1T48 does not impact apoptosis in MCF7 breast cancer cells (Online Resource 6). Thus, G1T48 selectively inhibits the growth of ER-positive, but not ER-negative, breast cancer cells.

### G1T48 inhibits estrogen signaling in endocrine-resistant breast cancer models

**Fig. 4:**
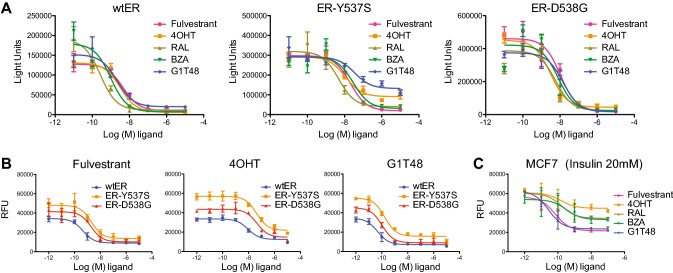
G1T48 inhibits ER signaling in models of endocrine therapy resistance in vitro **a** SKBR3 breast cancer cells were transfected with an estrogen-responsive reporter gene together with ER (wtER, ER-Y537S, or ER- D538G) expression plasmids prior to 18 h of treatment with 17β-estradiol (1.0 nM) and ER antagonists (10^–11^–10^–5^ M). Firefly and renilla luciferase activity were then assessed using dual luciferase reagent. Error bars indicate the SD of triplicate samples. **b** G1T48 inhibits cell growth mediated by endocrine refractory ER mutants. MCF7 cells expressing ER variants ER-D538G and ER-Y537S were treated for 7 days with doxycycline plus increasing dose of antiestrogens (10^–12^–10^–5^ M). Cellular proliferation was assessed by measuring DNA content (Hoechst stain) and is presented as relative fluorescence units. Error bars indicate the SD of triplicate samples. **c** G1T48 inhibits growth factor-mediated breast cancer cell growth. MCF7 cells were treated for 7 days with insulin (20 nM) plus increasing dose of anti-estrogens (10^–12^–10^–7^ M). Cellular proliferation was assessed by measuring DNA content (Hoechst stain) and is presented as relative fluorescence units. Error bars indicate the SD of triplicate samples

In addition to the upregulation of growth factor signaling, a second key mechanism of resistance to aromatase inhibition is newly described mutations in the ligand binding domain of ER [8, 9], mutations that result in reduced potency for 4OHT and fulvestrant as compared to wild-type (wt) receptor [44–50]. To assess the activity of G1T48 on endocrine refractory ER mutants, we utilized a reporter gene assay in ER-negative SKBR3 breast cancer cells transfected with ER expression vectors (wtER or the two most common ER mutants, ER-Y537S or ER-D538G) (Fig. 4a). G1T48 was found to be a potent and effective inhibitor of both wtER and ER-D538G transcription. As has been previously reported [49], all antiestrogens tested, including G1T48, demonstrated reduced potency against ER mutant transcriptional activity when compared to wtER (Online Resource 7). To further understand the significance of these results, MCF7 cells expressing doxycycline inducible wtER, ER-D538G, or ER-Y537S were engineered and G1T48 was evaluated for its ability to inhibit the ER-dependent growth of these cells. As has been previously reported for 4OHT and fulvestrant (and also confirmed here), G1T48 exhibited an increased GI_50_ in cells expressing the ER-Y537S and ER-D538G mutations when compared to wtER (Fig. 4b, Online Resource 7) [44, 45, 47–51]. Collectively, these data highlights that G1T48, like other SERDs, may be useful at targeting some mutant receptors, a potential that can be further evaluated clinically.

Dysregulated growth factor signaling has emerged as a primary mechanism of resistance to tamoxifen and AI therapy [10]. Activation of these signaling pathways can alter the pharmacology of compounds like tamoxifen, converting them from antagonists to agonists through phosphorylation of ER or its attendant coregulator proteins [4–7]. G1T48 and comparator SERMs and SERDs were evaluated for their ability to inhibit insulin-mediated MCF7 cell proliferation, a model for endocrine therapy resistance. Compounds with SERD activity, including G1T48, effectively blocked growth factor-mediated cell growth, while compounds with SERM activity (e.g., 4OHT) were less effective (Fig. 4c). These data together support the potential for G1T48 to have efficacy in the treatment of AI or tamoxifen-resistant breast cancers having growth factor pathway activation*.*

### Evaluation of the in vivo therapeutic efficacy of the SERD G1T48 and the CDK4/6 inhibitor lerociclib using estrogen-dependent and tamoxifen-resistant (TamR) breast cancer xenograft models

**Fig. 5:**
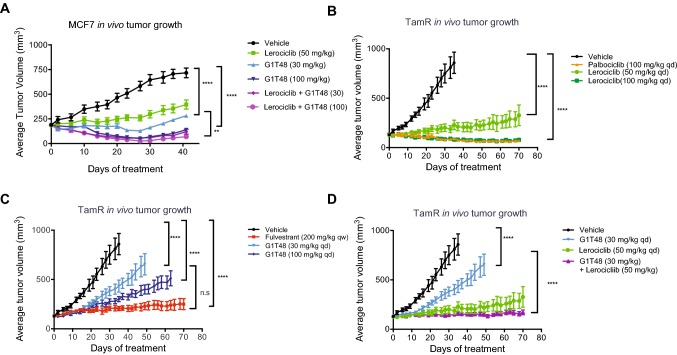
Combination strategy of G1T48 and the CDK4/6 inhibitor lerociclib inhibit estrogen-dependent and tamoxifen-resistant (TamR) breast cancer xenograft models in vivo ** a** Ovariectomized estrogen-treated female nu/nu mice bearing MCF7 xenograft tumors were randomized to treatment with vehicle, lerociclib (50 mg/kg) or G1T48 (30 or 100 mg/kg), alone or together, p.o. daily for 28 days. 2-way ANOVA comparison of average tumor volumes throughout treatment, followed by Bonferroni multiple comparison test, indicated significant tumor growth inhibition by all treatments, as well as increased response to the combination of G1T48 (30 mg/kg) and lerociclib (50 mg/kg). Error bars represent SEM. **b–d** Ovariectomized tamoxifen-treated female nu/nu mice bearing TamR xenograft were randomized to treatment with vehicle, palbociclib (100 mg/kg), lerociclib (50 or 100 mg/kg) (**b**), fulvestrant (200 mg/kg), or G1T48 (30 or 100 mg/kg) (**c**), with lerociclib and G1T48 being tested alone and in combination (**d**), p.o. daily for 28 days. 2-way ANOVA comparison of average tumor volumes throughout treatment, followed by Bonferroni multiple comparison test, indicated significant tumor growth inhibition by all treatments, as well as increased response to the combination of G1T48 (30 mg/kg) and lerociclib. Error bars represent SEM

We next evaluated the therapeutic potential of G1T48 in ER-positive primary and endocrine refractory breast cancer models in vivo. G1T48 was first assessed, as a monotherapy or in combination with the CDK4/6 inhibitor lerociclib, for its impact on the growth of naïve MCF7 xenograft tumors (Fig. 5a). Ovariectomized estrogen-treated female nu/nu mice bearing MCF7 xenograft tumors were randomized to treatment with vehicle, lerociclib (50 mg/kg), and/or G1T48 (30 or 100 mg/kg). G1T48 treatment demonstrated dose-dependent repression of tumor growth. Combination of lerociclib and G1T48 was more effective than either monotherapy, demonstrating an added benefit to using these agents together. End of study tumor volumes are presented in Online Resource 8.

The TamR xenograft model exhibits tamoxifen-stimulated growth that can be inhibited by compounds with SERD activity with added benefit observed upon combination with CDK4/6 inhibitors [52]. Therefore, ovariectomized tamoxifen-treated mice bearing TamR xenografts were randomized to treatment with lerociclib (50 mg/kg or 100 mg/kg), G1T48 (30 mg/kg or 100 mg/kg), fulvestrant (200 mg/kg), or CDK4/6 inhibitor palbociclib (100 mg/kg) as monotherapies or a combination of lerociclib (50 mg/kg) and G1T48 (30 or 100 mg/kg). In this model system, lerociclib demonstrated efficacy equivalent to that of the mechanistic clinical comparator palbociclib (Fig. 5b). G1T48 was found to demonstrate dose-dependent inhibition of TamR tumor growth (Fig. 5c) albeit with less efficacy than fulvestrant. Interestingly, G1T48 treatment resulted in greater downregulation of intratumoral ER levels than fulvestrant despite less efficient inhibition of tumor growth (Online Resource 8). Finally, combination of G1T48 with lerociclib, using suboptimal doses of each inhibitor, resulted in tumor growth inhibition significantly greater than that observed for either compound as monotherapy (Fig. 5d). End of study tumor volumes are presented in Online Resource 10. Mouse body weight was largely unaffected by the treatment regimens in this study as presented in Online Resource 11.

### Evaluation of the combined efficacy of lerociclib and G1T48 in a xenograft tumor model of resistance to estrogen deprivation in vivo

**Fig. 6:**
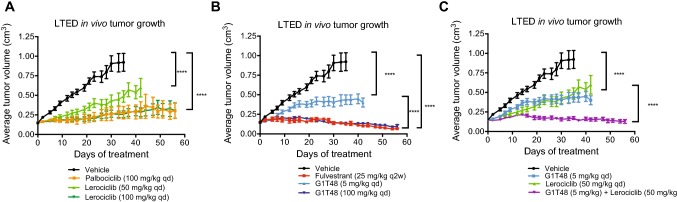
Combination strategy of G1T48 and the CDK4/6 inhibitor lerociclib in vivo in an estrogen-deprived xenograft model. Ovariectomized vehicle-treated female nu/nu mice bearing LTED xenograft were randomized to treatment with vehicle, lerociclib (50 or 100 mg/kg) or palbociclib (100 mg/kg) (**a**) or G1T48 (5 or 100 mg/kg) or fulvestrant (25 mg/kg) (**b**), alone or together (**c**), p.o. daily for 28 days. 2-way ANOVA comparison of average tumor volumes throughout treatment, followed by Bonferroni multiple comparison test, indicated significant tumor growth inhibition by all treatments, as well as increased response to the combination of G1T48 (30 mg/kg) and lerociclib. Error bars represent SEM

Although AIs have seen rapid adoption in the adjuvant setting*, *de novo and acquired resistance remains a persistent impediment to sustained clinical responses. We have developed an ER-positive model of aromatase resistance, termed long-term estrogen-deprived (LTED), to model this clinical situation [53]. In order to evaluate the combined efficacy of lerociclib and G1T48 in this model system, LTED xenograft tumors were orthotopically established in ovariectomized female nu/nu mice. When tumors measured 0.1–0.15 cm^3^ volume, G1T48 (5 mg/kg or 100 mg/kg) and lerociclib (50 mg/kg or 100 mg/kg) were administered alone and in combination, with fulvestrant (25 mg/kg) and palbociclib (100 mg/kg) included for comparison. As previously observed with the MCF7 parental and TamR models, G1T48 demonstrated dose-dependent inhibition of tumor growth (Fig. 6a). Additionally, the tumor growth inhibition after treatment with G1T48 and lerociclib was comparable to their clinical comparators (fulvestrant and palbociclib, respectively) (Fig. 6b). Combination of G1T48 with lerociclib suppressed tumor growth significantly compared to monotherapy (Fig. 6c) and resulted in tumor regression for a majority of tumors receiving the combined therapy regimen. End of study tumor volumes are presented in Online Resource 12.

### Evaluation of the combined efficacy of lerociclib and G1T48 in a patient-derived xenograft model harboring the ER-Y537S Mutation

**Fig. 7:**
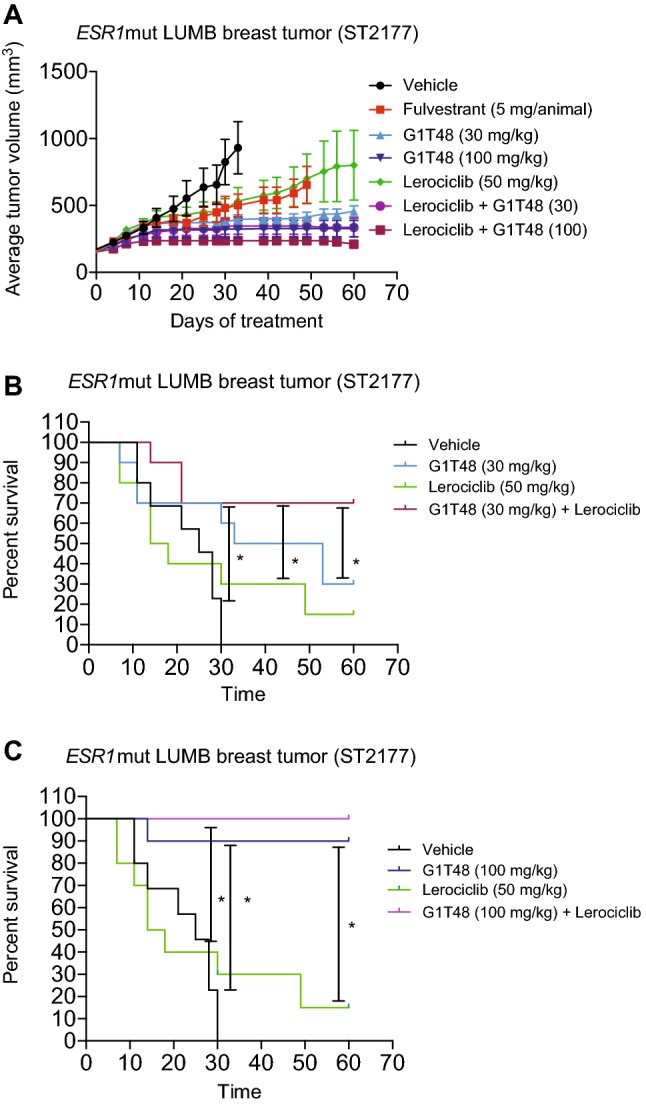
Evaluation of the combined efficacy of lerociclib and G1T48 in a Patient-Derived Xenograft Model harboring the ER-Y537S Mutation. **a** Female nu/nu mice were engrafted with a START Patient-Derived Xenograft Model (START-PDX) model, designated ST2177, harboring an ER-Y537S mutation. Mice were randomized to vehicle, fulvestrant (5 mg/animal), G1T48 (30 or 100 mg/kg), lerociclib (50 mg/kg), or the combination of G1T48 and lerociclib and treated for 60 days. **b**, **c** Kaplan–Meier analysis is presented as time for tumors to reach endpoint (2.5 times original tumor volume). *Kaplan–Meier analysis followed by a Mantel–Cox test for significance demonstrated significantly greater tumor growth delay for these comparisons using an adjusted Bonferroni threshold of *p* < 0.012. Error bars represent SEM

As described above, mutations in the LBD of *ESR1* are an emerging mechanism of resistance to AIs. The efficacy of G1T48, as a mono- and combination therapy with lerociclib, was evaluated using a Patient-Derived Xenograft (PDX model) harboring the ER-Y537S mutation (Fig. 7a). Female athymic nu/nu mice were implanted with the ST2177 LUMB PDX tumor [33] and following treatment with G1T48, a dose-dependent decrease in tumor growth was observed. Intriguingly, G1T48 alone (30 and 100 mg/kg) or in combination with lerociclib was more efficacious than fulvestrant (Fig. 7a). Survival curve analysis demonstrated that the combination of 30 mg/kg G1T48 with lerociclib was more effective than monotherapy using either drug alone (Fig. 7b, c). Taken together these data highlight that G1T48 is either comparable or superior to fulvestrant in several models of endocrine therapy resistance, demonstrating its potential as a therapeutic agent.

## Discussion

Targeting ER activity using therapies that directly oppose the mitogenic action of estrogen or that block estrogen synthesis is a proven strategy for the treatment and prevention of breast cancer. In locally advanced or metastatic disease, resistance to these therapies frequently emerges within two years, at which time treatment options are severely limited. Fulvestrant, a potent ER antagonist and downregulator, was initially approved for the treatment of endocrine therapy-resistant disease and more recently as first-line therapy for advanced ER-positive, HER2-negative breast cancer not previously treated with endocrine therapy. However, despite promising preclinical activity, the poorly controlled pharmacokinetics of fulvestrant remains a significant barrier to prolonged clinical efficacy. Clinical trials comparing high-dose (500 mg) to low-dose (250 mg) fulvestrant demonstrated superiority for the 500 mg dose in both first- and second-line settings, suggesting that increased target engagement can improve the outcome of ER degradation therapy [54, 55]. However, given its intramuscular route of administration, continued improvements in the clinical response to fulvestrant by further dose escalation appear unlikely. Therefore, development campaigns in this area have focused on the identification of orally bioavailable SERDs. The most active SERDs share common chemical features: either (a) a steroidal backbone (e.g., fulvestrant, RU58,668) or (b) an acrylic acid side chain (GW7604, GDC-0810, AZD9496) [29, 31, 32, 40, 56, 57]. Additional ER antagonists with novel chemical structures have also been reported to exhibit SERD properties [35, 36, 58], but none has yet gained FDA approval and some have been discontinued due to adverse effects or for undisclosed reasons [29–36, 40, 56]. We have identified a novel, orally bioavailable SERD, G1T48, that contains both a steroidal backbone and an acrylate side chain. G1T48 binds ER with low nanomolar affinity, inhibits estrogen-mediated target gene expression and breast cancer cell growth, and importantly blocks the tumor promoting effects of ER in both naïve and endocrine therapy-resistant animal models of breast cancer. Similar to AZD9496 and GDC-0810, G1T48 has good pharmacokinetic properties and maintains a more favorable side-effect profile compared to those reported for AZD9496 [56, 59, 60].

A hallmark feature of fulvestrant differentiating it from compounds like tamoxifen is that fulvestrant is a true antagonist with no agonist activity regardless of tissue context. By contrast, tamoxifen is a Selective Estrogen Receptor Modulator (SERM), demonstrating robust antagonist activity in the breast, but mimicking the agonist effect of estrogen in bone, the endometrium, and serum lipid profiles [61, 62]. This mechanistic difference between tamoxifen and fulvestrant can also be observed in breast cancer cells, where transcriptional profiling studies revealed that tamoxifen can regulate a subset of genes in a similar manner to estradiol. Our ER target gene regulation studies confirm the agonist activity of tamoxifen, with stimulation of *SDK2*, *AGR2*, and *RAPGEL1* expression similar to the effect of estrogen treatment [42]. Compounds with SERD activity such as fulvestrant and AZD9496 did not increase these transcripts, consistent with a lack of agonist potential (i.e., pure antagonism). The transcriptional profile in breast cancer cells of G1T48 is most similar to fulvestrant and other SERDs. Interestingly, our studies revealed that there were modest differences in the transcriptional profiles even among the pure antagonist class of compounds, suggesting that they might engender different receptor conformations. The impact of these differences in ER target gene activation remain to be explored but could suggest that cross-resistance between different classes of SERDs can be avoided. Recent studies have indicated that in addition to receptor degradation, ER mobility is differentially impacted by sub-classes of SERMs and SERDs, and that compounds impeding mobility are more efficacious antagonists [35]. The impact of G1T48 on ER mobility is not currently known; however, our studies establish that G1T48 has very low intrinsic ER agonist activity.

Acquired resistance to endocrine therapy is complex and multifactorial; however, mutations in the *ESR1* gene that result in ligand-independent receptor activity have emerged as a potential mechanism to account for approximately 30–40% of resistant disease following AI treatment [8, 44–46, 48–51]. It is significant, therefore, that G1T48 was found to suppress both the ligand-independent cell growth and transcriptional activity attributed to the two most prevalent endocrine refractory ER mutants, ER-Y537S and ER-D538G. Intriguingly, in contrast to the reconstituted transactivation assay in SKBR3, G1T48 was found to efficiently inhibit the growth of MCF7 cells engineered to overexpress the ER-Y537S variant. Cell context may contribute to this discrepancy; differential cofactor expression patterns in the two cell lines and/or the presence of endogenous wtER in MCF7 cells may influence G1T48 efficacy.

Long-term estrogen deprivation leading to a state of estrogen hypersensitivity is another means to model aromatase inhibitor therapy resistance. We have developed a new model of resistance to estrogen deprivation without ER mutation [53]. Using this model system, treatment with low-dose G1T48 (5 mg) resulted in incomplete tumor growth inhibition, while high-dose G1T48 (100 mg) as monotherapy resulted in tumor regression in the majority of animals, similar to fulvestrant, demonstrating the effectiveness of SERD therapy in this setting of resistant disease.

The combination of SERDs with CDK4/6 inhibitors has been evaluated clinically, most recently in the PALOMA-3 trial comparing the co-administration of the CDK4/6 inhibitor palbociclib (Ibrance^®^) with fulvestrant to fulvestrant alone. The results of this study demonstrated an overall survival benefit (median survival 34.9 months compared to 28 months) and a significant progression free survival rate (9.5 months vs 4.6 months) for the combination arm [17, 20]. These noteworthy improvements led to the 2016 FDA approval of palbociclib and fulvestrant combination therapy for ER-positive, HER-2- negative breast cancers progressing on other endocrine therapies. Further trials (MONALESSA-3 (NCT02422615) and MONARCH-2 (NCT02107703) have also demonstrated the utility of administering other CDK4/6 inhibitors with fulvestrant to improve patient outcomes [17–20]. The increased efficacy observed for the combination of G1T48 and lerociclib, as compared to monotherapy administration, in multiple in vivo breast cancer models sensitive or refractory to endocrine therapy treatment supports the potential utility of this regimen as an intervention in multiple stages of breast cancer treatment. Furthermore, lerociclib has been shown to promote less myelosuppression than palbociclib [25, 26]. Collectively, these data indicate that G1T48 has the potential to be an efficacious oral antineoplastic agent in ER+ breast cancer.

## Electronic supplementary material

Below is the link to the electronic supplementary material.

Supplementary file1 (DOCX 18858 kb)
