# Supplementary material for: G1T48, an oral selective estrogen receptor degrader, and the CDK4/6 inhibitor lerociclib inhibit tumor growth in animal models of endocrine-resistant breast cancer
Source: Breast Cancer Res Treat. 2020 Mar 4;180(3):635–46. doi: 10.1007/s10549-020-05575-9 (PMC7103015; doi:10.1007/s10549-020-05575-9)
Supplement: Supplementary file 1 — Supplementary file1 (DOCX 18858 kb) [file 10549_2020_5575_MOESM1_ESM.docx]

**Online Resource 1: Supplemental Methods**

**RNA Profiling:** (Continued from RNA analysis) For ER gene signature, the data were first normalized to the vehicle control within each gene. To avoid signal strength bias, the data were standardized using the following equation; Ζ=Χ-μ/σ where Χ is the normalized signal, μ is the average signal for all conditions within a gene and σ is the standard deviation. The data are then clustered with the Ward hierarchical clustering method using JMP (SAS). The hierarchical clustering dendogram is ordered by the first principle component.

**In-Cell Western**: MCF7 cells were plated in DMEM/F12 supplemented with 8% charcoal dextran treated FBS in 96-well clear bottom black plates (25K cells/well). After 48 hour incubation, cells were treated with hormone (dose response; 10^-11^ to 10^-5^ M) for 24 hours. Cells were fixed with formaldehyde (3.7%), permeabilized using PBS 0.1% TRITON X-100, and incubated with anti-ER antibody (HC-20, Santa Cruz, 1:1000) overnight. Cells were washed with PBS 0.1% Tween, and stained with 2nd antibody (Biotium CF770 goat anti-rabbit, 1:2000). ER protein expression was assessed using the LI-COR Odyssey imaging system. DRAQ5 (DNA stain, 1:10,000, Thermo Scientific) was used to normalize ER protein expression. Data are reported as percent ER remaining after drug treatment.

**Radioactive Binding Assay**: MCF7 cells were plated in DMEM/F12 supplemented with 8% charcoal dextran treated FBS in 24 well plates (500K cells/well). After 48 hour incubation, cells were treated with 0.1 nM ^3^H-17β-E2 (PerkinElmer Catalog number NET317, lot number 2526124) and competitor ligand (dose response; 10^-11^ to 10^-6^ M) for 2 hours. Cells were washed 2X with DMEM/F12 supplemented with 8% charcoal dextran treated FBS and 1X with 1X Phosphate Buffered Saline (PBS). Cells were lysed with 200 µL of Lysis buffer (2%S SDS, 10% glycerol, 10mM Tris-Cl pH 6.8). Cell lysates were diluted with 300 µL of 10mM of Tris- HCl pH 8.0. Scintillation vials were prepared with 3 mL of CytoScint. 300 µL of lysate was added to respective scintialltion vials. Samples were read on a Beckman LS 6000SC Scintillation counter for 1 minute per sample.

**Chromatin Immunoprecipitation (ChIP):** MCF7 cells were grown to 90% confluence in DMEM/F12 supplemented with 8% charcoal dextran treated FBS for 3 days, at which time cells were treated with ligand for 90 minutes and subjected to ChIP analysis. Each plate of cells was cross-linked with 1% formaldehyde PBS solution for a maximum 10 minutes and quenched with 125mM glycine solution containing 5mg/ml bovine serum albumin (BSA) for 5 minutes. Cells were then rinsed once and harvested with ice cold PBS, pelleted at 8000 rpm for 30 seconds at room temperature and snap frozen for storage at -80ºC. All solutions were supplemented with 10mM sodium butyrate and protease inhibitors. Cell pellets were thawed on ice, and then resuspended in 10mL Lysis Buffer 1 (50mM HEPES pH 7.5, 140 nM NaCl, 1 mM EDTA 10% Glycerol, 5% NP-40, 2.5% Triton X-100, protease inhibitor cocktail). These solutions were incubated for 10 minutes rocking at 4°C. Samples were then spun down at 2000 rpm for 4 minutes, supernatant removed, resuspended in 10mL Lysis Buffer 2 (10mM Tris pH 8.0, 200 nM NaCl, 1 mM EDTA pH 8.0, 0.5 mM EGTA pH 8.0, protease inhibitor cocktail), and then incubated for 5 minutes while rocking at 4°C. Samples were then spun again at 2000 rpm for 4 minutes, supernatant removed, and resuspended in 1mL Lysis Buffer 3 (0.1% NaDeoxycholate, 0.5% N-laurylsarcosine, 1 mM EDTA, 0.5 mM EGTA, 10 mM Tris pH 8.0, 100 mM NaCl, protease inhibitor cocktail). Cell lysates then were sonicated using the Covaris S220 instrument according to the manufacturers’ instructions. Triton X-100 was then added to the sheared chromatin samples to make a final concentration of 1%, and samples then centrifuged at 13000 rpm for 10 minutes at 4°C. Supernatant was then transferred to a new tube and sheared chromatin was diluted using Dilution Buffer (20 mM Tris [pH 8.0], 150 mM NaCl, 2 mM EDTA, 1% Triton X-100). ER ChIP was performed by incubating sheared, diluted chromatin with 5 µg of D12 anti-ER antibody. Antibodies were allowed to bind overnight at 4°C while rotating and then captured on protein A/G magnetic beads (Pierce, Cat#: 88802) which had been previously washed three times with 5 mg/mL BSA. After 45 min of incubation with the beads, the immunoprecipitates were washed twice with Wash Buffer A (50 mM HEPES pH 7.8, 500 mM NaCl, 1 mM EDTA, 1% Triton X-100, 0.1% Deoxycholate, 0.1% SDS, protease inhibitor cocktail), twice with Buffer B (20 mM Tris pH 8.0, 1 mM EDTA, 0.5% NP40, 0.5% Na Deoxycholate, 0.25 M LiCl), and twice with TE Buffer (20 mM Tris pH 8.0, 2 mM EDTA). Following washes, precipitates were eluted in Elution Buffer (50 mM Tris pH 8.0, 1 mM EDTA, 1% SDS). Crosslink reversal was performed by addition of 21 µL 5 M NaCl to each sample and incubated at 65°C over-night. 4 µL 0.5 M EDTA and 1 µL 20 mg/mL proteinase K was then added to each sample and incubated at 42° C for 1 hour. DNA was then purified using the Qiagen PCR purification kit (Cat#: 28104) according to manufacturer’s instructions. ER recruitment was then assessed via qPCR.

**Transcriptional Reporter Assays:** SKBR3 or CV1 cells were transfected with NR expression constructs, reporter genes (3XERE- TATA-LUC or MMTV-Luc) and renilla luciferase (toxicity assessment) using lipofectin reagent. Cells were treated with respective ligand for 24 hours. Cells were lysed and protein extracts were assessed for firefly and renilla luciferase activity using dual luciferase reagent (DLR).

**Apoptosis Assay:** MCF7 cells were plated in 6- well plates in DMEM/F12 supplemented with 8% charcoal dextran treated FBS and treated with DMSO, 1.0 µM of G1T48 or 1.0 µM of fulvestrant for 5 days. Control cells were grown in tandem and then treated with 1.0 nM Docetaxal 16 hours before collection. Cells and supernatant were harvested in ice cold PBS, centrifuged, and resuspended in 100 µL Annexin Binding Buffer (10 mM HEPES, 140 mM NaCl, and 2.5 mM CaCl_2_, pH 7.4). Annexin V conjugate (5µL; Invitrogen Catalog number A13201) and SYTOX (0.2 uL in 4.8 uL of annexin-binding buffer; Thermo-Fisher Catalog number S34859) were added for 15 minutes. Stained cells were counted using a Accuri C6 Flow Machine, and data was analyzed using FloJo software.

**Doxycycline Inducible Cell Line Generation:** Doxycycline inducible cell lines were engineered from parental MCF7 cells in the laboratory of Dr. Donald McDonnell. Specifically, MCF7 cells were first infected with (pLenti CMV RTTA3, blastocidin resistant) followed by (pLenti CMV TRE3G, puromycin resistant) using the following protocol. TS293 cells were transfected with VSVG envelope vector and PsPAX2 (all gifts from Kris Wood, Duke University School of Medicine) and the viral construct of interest using Fugene 6 (Promega E2691) per the manufacturer protocol. Media was removed and replaced with DMEM containing 30% FBS 18 hours after transfection. Cells were allowed to produce virus for 2 days and then media was collected. Viral media was filtered and 4 µg/mL of polybrene was added. Viral containing media was added to MCF7 cells that had been split 1:3 24 hours prior. After 48 hours, antibiotic selection was added and cells were selected. The cells used in these experiments are maintained in DMEM/ F12 media supplemented with blastocidin and puromycin to maintain the constructs. Proliferation assays were performed as described in the methods section

**MCF7 Naïve Tumor Studies:** South Texas Accelerated Research Therapeutics (START, San Antonio, Texas) evaluated antitumor activity of G1 Therapeutics test (G1T48 and lerociclib) agents in a Cell-Based Xenograft (CBX) model, MCF7, representing human ER positive breast cancer, in immune deficient mice. Female athymic nude mice (Crl:NU(NCr)-Foxn1^nu^) at 6-12 weeks of age were implanted subcutaneously with cultured MCF7 cells. Estrogen was supplemented via the drinking water to the animals. The study was initiated at a mean tumor volume of approximately 150-250 mm^3^. G1T48 and lerociclib were formulated in 50 mM citrate buffer, pH 4.3 and dosed at 10 mL/kg, PO/ qd x 28. Data collection endpoint for this study was completed on day 62

**TamR Tumor Studies:** All procedures were approved by the Duke University Institutional Animal Care and Use Committee (IACUC) prior to initiating the experiment. 120 female nu/nu mice (~6 weeks of age) were ovariectomized under anesthesia (isoflurane) and in the same procedure implanted sc (scapular region) with tamoxifen (Tam) treatment pellets (5 mg/60 days, ~3.3 mg/kg/d continuous release, Innovative Research of America) 24-48 hours prior to having an ~8mm^3^ section of tamoxifen-resistant (TamR) tumor tissue engrafted orthotopically (right axial mammary fat pad) under anesthesia. Tumors were measured 3X weekly, concurrent with weight and behavior monitoring, until tumors reached ~0.1-0.15 cm^3^ volume (*l* x *w^2^* x 0.5). Mice were then randomized (n = 8-10) to treatment with: Vehicle, fulvestrant (200 mg/kg), (palbociclib - 100 mg/kg), G1T48 (30 or 100 mg/kg), or the combination of G1T48 + lerocicilib. Vehicle used in this study was 9% PEG 400/0.5% PVP/0.5% Tween 80/ 0.05% CMC for orally gavaged compounds. Fulvestrant was formulated in 5% DMSO/95% NF grade corn oil.Treatments were administered weekly (fulvestrant) or daily as indicated for 10 weeks, and tumor measurement and weight monitoring continued as above throughout that time. 2-3 hours after the final treatment, animals were euthanized by CO_2_ exposure, followed immediately by cardiac puncture for blood collection and secondary method decapitation. Plasma and tumor tissues were cryopreserved for future analysis. Frozen tissues were pulverized prior to protein extraction in RIPA buffer (50 mM Tris, pH 8, 150 mM NaCl, 1% NP-40, 0.5% deoxycholate, 0.02% SDS, 1 mM EDTA). 25 µg of cleared extracts were resolved by SDS-PAGE prior to transfer to PVDF membrane and immunoblot analysis by standard methods. Bands detected were quantitated using ImageJ per standard methods (<http://lukemiller.org/index.php/2010/11/analyzing-gels-and-western-blots-with-image-j/>).

**LTED Tumor Studies:** All procedures were approved by the Duke University Institutional Animal Care and Use Committee (IACUC) prior to initiating the experiment. 120 female nu/nu mice (~6 weeks of age) were ovariectomized under anesthesia (isoflurane) 24-48 hours prior to having an ~8mm^3^ section of long-term estrogen deprivation (LTED) tumor tissue engrafted orthotopically (right axial mammary fat pad) under anesthesia. Tumors were measured 3X weekly, concurrent with weight and behavior monitoring, until tumors reached ~0.1-0.15 cm^3^ volume (*l* x *w^2^* x 0.5). Mice were then randomized (n = 8-10) to treatment with: Vehicle (10 mM citrate buffer + oral vehicle 10:5:85 (below)), fulvestrant (25 mg/kg fulvestrant), palbociclib (100 mg/kg) G1T48 (5 or 100 mg/kg), lerociclib (50 or 100 mg/kg), or combination treatments. Treatments were administered bi-weekly (fulvestrant) or daily as indicated for up to 8 weeks, and tumor measurement and weight monitoring continued as above throughout that time. 2-3 hours after the final treatment, animals were euthanized by CO_2_ exposure, followed immediately by cardiac puncture for blood collection and secondary method decapitation. Plasma and tumor tissues were cryopreserved for future analysis.

**PDX Tumor Study**: South Texas Accelerated Research Therapeutics (START) evaluated antitumor activity of G1 Therapeutics test agents, G1T38 and G1T48, in a START Patient-Derived Xenograft (START-PDX) model, designated ST2177, representing human *ESR1* mutant, Y537S, ER+ breast cancer. Female athymic nude mice (Crl:NU(NCr)-Foxn1^nu^) at 6-12 weeks of age were implanted subcutaneously with cultured MCF7 cells. The study was initiated at a mean tumor volume of approximately 150-250 mm^3^. G1T48 and lerociclib were formulated in 50 mM citrate buffer, pH 4.3 and dosed at 10 mL/kg, PO/ qd x 28. Fulvestrant was administered through subcutaneous injection. Data collection endpoint for this study was completed on day 33. In order to account for tumor growth outliers, the mouse having the largest final tumor growth measurement in each group was excluded from the tumor growth plots. All animals were included in the survival curve analysis.

**Statistics:** Tumor growth data were subjected to exponential growth curve analysis constrained to share an initial value, and to two-way ANOVA analysis followed by Bonferroni multiple comparison test. Significant difference as compared to the vehicle treated control (p<0.05) was detected for multiple groups at several time points (indicated on graphs). Groups showed equivalent variance (10-15% with normal distribution) throughout all time points, justifying the statistical analyses that were selected. % change in tumor volume was calculated per the following equation: % change = (final tumor volume/initial tumor volume) – 1. Comparison of % change in tumor volume between groups was done using a one-way ANOVA followed by Holm-Sidak multiple comparison test.

**Online Resource 2: ER degradation IC_50_ values**

| **Compound** | **IC_50_ (M)** |
| --- | --- |
| Fulvestrant | 2.74E-10 |
| RU 58668 | 2.02E-11 |
| Tamoxifen | 4.46E-08 |
| 4-hydroxytamoxifen | 1.00E-10 |
| GW5638 | 3.11E-07 |
| GW7604 | 4.20E-10 |
| Raloxifene | 5.94E-11 |
| Bazedoxifene | 5.69E-10 |
| GDC-0810 | 9.49E-11 |
| AZD9496 | 1.08E-11 |
| G1T48 | 3.30E-11 |
| Lasofoxifene | 1.89E-11 |

**Online Resource 3: Radioactive Binding Assay IC_50_ values**

| **Compound** | **IC_50_ (M)** |
| --- | --- |
| Estradiol (E2) | 6.62E-11 |
| Fulvestrant | 2.99E-09 |
| Raloxifene | 2.26E-10 |
| Bazedoxifene | 7.27E-10 |
| G1T48 | 8.54E-10 |

**Online Resource 4: Impact of G1T48 on steroid hormone receptor transcriptional activity**

CV-1 cells were transfected with AR, GR, MR, or PR expression constructs, along with the MMTV-Luc reporter gene, and *Renilla*-Luc. Cells were treated with R1881, dexamethasone, aldosterone, or progesterone (0.1 nM) plus the indicated dose of antagonist for 24 hours. Dual-luciferase values were measured and data is presented as light units. Error bars indicate the SD of triplicate samples.

**Online Resource 5: Breast cancer proliferation GI_50_ (M) values**

| **Compound** | **MCF7** | **BT474** | **ZR-75-1** | **MDA-MB-436** |
| --- | --- | --- | --- | --- |
| Fulvestrant | 8.48E-10 | 1.15E-08 | 7.21E-08 | >1.0E-05 |
| 4-hydroxytamoxifen | 3.58E-09 | 9.99E-07 | 1.02E-08 | >1.0E-05 |
| GDC-0810 | 1.82E-09 | 1.20E-07 | 1.17E-07 | >1.0E-05 |
| AZD9496 | 4.58E-11 | 2.68E-09 | 2.28E-09 | >1.0E-05 |
| G1T48 | 2.57E-10 | 2.21E-08 | 9.62E-08 | >1.0E-05 |

**Online Resource 6: Impact of G1T48 on breast cancer cell apoptosis**

MCF7 cells were treated with DMSO, 1µM of G1T48 or 1µM of fulvestrant for 5 days. Control cells were grown in tandem and then treated with 1 nM Docetaxal 16 hours before collection. Cells and supernatant were harvested and stained using Annexin V and SYTOX. Cells that stained double positive are considered to be in late apoptosis.

**Online Resource 7: ER mutant and wtER transcriptional IC_50_ (M) values**

| **Compound** | **wtER** | **ER-Y537S** | **ER-D538G** |
| --- | --- | --- | --- |
| Fulvestrant | 4.49E-09 | 2.02E-08 | 1.05E-08 |
| 4-hydroxytamoxifen | 3.25E-09 | 1.10E-08 | 3.11E-09 |
| Raloxifene | 2.73E-10 | 4.79E-09 | 5.76E-09 |
| Bazedoxifene | 9.79E-10 | 2.90E-08 | 7.97E-09 |
| G1T48 | 2.14E-09 | 3.45E-08 | 1.47E-08 |

**Online Resource 8: G1T48, alone or in combination with lerociclib, inhibits the growth of estrogen-dependent MCF7 xenograft tumors**

Ovariectomized estrogen-treated female nu/nu mice bearing MCF7 xenograft tumors were randomized to treatment with vehicle, G1T38 (50 mg/kg) or G1T48 (30 or 100 mg/kg), alone or together, p.o. daily for 41 days. 2-way ANOVA comparison of average tumor volumes throughout treatment, followed by Bonferroni multiple comparison test, indicated significant tumor growth inhibition by all treatments, as well as increased response to the combination of G1T48 (30 mg/kg) and G1T38.

**Online Resource 9: Analysis of intratumoral *ESR1* protein levels in harvested tumor tissue (TamR)**

Analysis of intratumoral *ESR1* protein levels in harvested tumor tissue (TamR): Preserved tumor tissues from fulvestrant treatment groups featured in Figure 6 were processed as described (methods) and analyzed by immunoblot. Bands detected were quantitated (ImageJ) and normalized within each blot to the average expression detected for triplicate vehicle samples (the same 3 vehicle samples were included on each separate blot, indicated by the red boxes).

**Online Resource 10: G1T48 and lerociclib both inhibit the growth of tamoxifen resistant (TamR) xenograft tumors.**

TamR change in tumor volume with combined G1T48/lerociclib treatment regimen. Relative change in tumor volume after 10 weeks of treatment was calculated for each tumor treated with G1T48 (30 mg/kg) + lerociclib (50 mg/kg).

**Online Resource 11: Impact of G1T48 on mouse body weight throughout TamR tumor study**

Mouse body weight throughout the course of the TamR Tumor Study. The following mice had weights that were outliers compared to others in that group. Mouse A in the lerociclib cohort was euthanized with ideopathic lymphoma.

Mouse B (as seen over the course of several days and displayed in the black circle) in combined cohort struggled to learn lixit water spigot and required supportive care until it was able to acquire water on its own. Mouse C in high dose G1T48 cohort suffered a gavage error and was euthanized.

**Online Resource 12: G1T48 and lerociclib, alone or in combination, inhibit the growth of long-term estrogen deprived (LTED) xenograft tumors**

Comparison of % change in tumor volume in response to treatment in LTED. Comparison were made between groups of the % change in tumor volume (calculated as final volume/initial volume – 1) for individual tumors. Relevant comparisons were made between SERD treatments (A), CDK4/6 inhibitor treatments (B), and single and combined treatment arms featuring the 50 mg/kg (C) or 100 mg/kg (D) dose of lerociclib.
